# The organ-specific expression of terpene synthase genes contributes to the terpene hydrocarbon composition of chamomile essential oils

**DOI:** 10.1186/1471-2229-12-84

**Published:** 2012-06-08

**Authors:** Sandra Irmisch, Sandra T Krause, Grit Kunert, Jonathan Gershenzon, Jörg Degenhardt, Tobias G Köllner

**Affiliations:** 1 Institute of Pharmacy, Martin Luther University, Hoher Weg 8, Halle 06120, Germany; 2 Max Planck Institute for Chemical Ecology, Hans-Knöll-Strasse 8, Jena 07745, Germany; 3 Current address: Max Planck Institute for Chemical Ecology, Hans-Knöll-Strasse 8, Jena 07745, Germany

## Abstract

**Background:**

The essential oil of chamomile, one of the oldest and agronomically most important medicinal plant species in Europe, has significant antiphlogistic, spasmolytic and antimicrobial activities. It is rich in chamazulene, a pharmaceutically active compound spontaneously formed during steam distillation from the sesquiterpene lactone matricine. Chamomile oil also contains sesquiterpene alcohols and hydrocarbons which are produced by the action of terpene synthases (TPS), the key enzymes in constructing terpene carbon skeletons.

**Results:**

Here, we present the identification and characterization of five TPS enzymes contributing to terpene biosynthesis in chamomile (*Matricaria recutita*). Four of these enzymes were exclusively expressed in above-ground organs and produced the common terpene hydrocarbons (−)-(*E*)-β-caryophyllene (MrTPS1), (+)-germacrene A (MrTPS3), (*E*)-β-ocimene (MrTPS4) and (−)-germacrene D (MrTPS5). A fifth TPS, the multiproduct enzyme MrTPS2, was mainly expressed in roots and formed several Asteraceae-specific tricyclic sesquiterpenes with (−)-α-isocomene being the major product. The *TPS* transcript accumulation patterns in different organs of chamomile were consistent with the abundance of the corresponding TPS products isolated from these organs suggesting that the spatial regulation of *TPS* gene expression qualitatively contribute to terpene composition.

**Conclusions:**

The terpene synthases characterized in this study are involved in the organ-specific formation of essential oils in chamomile. While the products of MrTPS1, MrTPS2, MrTPS4 and MrTPS5 accumulate in the oils without further chemical alterations, (+)-germacrene A produced by MrTPS3 accumulates only in trace amounts, indicating that it is converted into another compound like matricine. Thus, *MrTPS3*, but also the other *TPS* genes, are good markers for further breeding of chamomile cultivars rich in pharmaceutically active essential oils.

## Background

Chamomile (*Matricaria recutita* [L.] Rauschert, Asteraceae) is one of the oldest and agronomically most important medicinal plant species in Europe. It originates from southeastern Europe and western Asia, but is nowadays cultivated throughout the world. The essential oil of chamomile flowers has significant antiphlogistic [1], spasmolytic [2] and antimicrobial [3] activity and is therefore used for several pharmaceutical, nutritional and cosmetic applications. The pharmaceutically active components of the flower oil are chamazulene, a degradation product spontaneously formed during steam distillation from the sesquiterpene lactone matricine, several bisabolol-type sesquiterpenes ((−)-α-bisabolol, bisabolol oxides), flavonoids and two en-in-dicycloethers [4-6] with chamazulene and the bisabolols being the main active constituents [7]*.*

The qualitative and quantitative terpene composition of the flower oil varies among different chamomile cultivars [6,8] and is dependent on the developmental stage and the cultivation conditions of the plant [4,9,10]. Besides flowers, chamomile roots and shoots are also rich in essential oil. However, in contrast to the flower oil which is mainly produced in glandular trichomes, these oils accumulate in schizogenous oil passages and oil cells and are dominated by sesquiterpene hydrocarbons and alcohols like (*E*)-β-farnesene and spathulenol, respectively [5,6].

Terpenes are produced by the action of terpene synthases (TPSs), which convert the ubiquitous prenyl diphosphates, geranyl diphosphate (GPP), farnesyl diphosphate (FPP) and geranylgeranyl diphosphate (GGPP) into the respective mono-, sesqui- and diterpene skeletons. Common to all terpene synthases is the formation of highly reactive carbocationic intermediates which can undergo a great variety of rearrangements resulting in a huge number of different terpene structures (reviewed in [11]). Many terpene synthases are multiproduct enzymes producing more than one compound from their substrate. For example, the recently reported enzyme MtTPS5 from *Medicago truncatula* forms a complex mixture of 27 sesquiterpenes [12]. Thus, the complex terpene blends of plants are often produced by only a limited number of multiproduct TPS enzymes [13-17].

Despite the pharmaceutical and economic importance of chamomile essential oil, little is known about the biosynthesis of its major constituents in chamomile. Thus, we started to investigate enzymes responsible for terpene biosynthesis in this plant species. Here, we report the identification and characterization of five terpene synthases involved in essential oil production. QRT-PCR analysis revealed organ-specific expression patterns of *TPS* genes which are consistent with the abundance of enzyme products in the respective plant organs. A (+)-germacrene A synthase (MrTPS3) is most likely a key enzyme in the biosynthesis of the pharmaceutical active sequiterpene lactone matricine.

## Results

### The terpene composition of chamomile essential oils isolated from different plant organs

To study terpene biosynthesis in chamomile, we used the German cultivar ‘Bodegold’ which was reported to be rich in the total amount of flower essential oil [7]. Since the terpene composition in the different plant organs of this cultivar has not been comprehensively described, a detailed terpene analysis including ray florets, disk florets, leaves, stems and roots was performed (Table 1). The ray florets and disk florets showed an identical blend of terpenes (Table 1). The total amount of terpenes in the disk florets, however, was approximately four times higher than in the ray florets (disk florets, 1098 ± 180 μg/g fw; ray florets, 308 ± 46 μg/g fw). Both flower types produced the sesquiterpenes bisabolol oxide A (disk florets, 28.1% of total terpenes; ray florets, 32.0%), bisabolol oxide B (disk florets, 17.6%; ray florets, 28.4%), α-bisabolol (disk florets, 18.4%; ray florets, 21.4%), (*E*)-β-farnesene (disk florets, 21.6%; ray florets, 6.9%) and (−)-germacrene D (disk florets, 5.3%; ray florets, 2.0%) as major components. Additionally, 15 mono- and sesquiterpenes could be identified as minor compounds. In contrast to flowers, the leaf terpene blend was dominated by the sesquiterpene hydrocarbons (*EE*)-α-farnesene (54.1%), (−)-germacrene D (22.4%), bicyclogermacrene (6.4%) and β-selinene (4.8%). Monoterpenes were only found in smaller amounts. α-Bisabolol and the bisabolol oxides could not be detected in the leaves. The terpene composition of the stems was nearly identical to that of the leaves but the quantity of some of the compounds differed significantly. The major compound in the stems was identified as the sesquiterpene hydrocarbon (*E*)-β-farnesene (71.8%). In the roots, (*E*)-β-farnesene also dominated the terpene blend (63.5%). Besides (*E*)-β-farnesene, the unusual triquinane-type sesquiterpene, α-isocomene (10.8%), and the monoterpenoid ester geranyl valerate (10.7%) were also detected in roots. In contrast to the above-ground organs of the plant, the roots produced no monoterpenes.

**Table 1:** **Amount of terpenoids (μg/g fresh weight) in the different plant parts of*Matricaria recutita*cultivar Bodegold and the statistical significance of their distribution**

**Compound**	**Ray florets**	**Disk florets**	**Leaves**	**Stem**	**Roots**	**F or*χ²***	**p**
*Total terpenoids*	*307.75 ± 45.65 a*	*1097.66 ± 179.53 b*	*527.61 ± 173.54 a*	*515 ± 83.48 a*	*325.98 ± 28.98 a*	*7.207*	*0.003*
α-pinene*	0.22 ± 0.19 ab	0.62 ± 0.29 a	0.02 ± 0.02 b	0.01 ± 0.01 b	0 b	9.768	<0.001
sabinene*	5.37 ± 5.09 a	12.68 ± 12.04 a	0 b	0.01 ± 0.01 b	0 b	11.427	<0.001
myrcene*	2.03 ± 1.68 a	0.82 ± 0.28 a	0.19 ± 0.08 a	0.23 ± 0.09 a	0 b	13.403	<0.001
limonene*	0.28 ± 0.21 ab	0.59 ± 0.19 a	0 b	0.03 ± 0.02 b	0 b	4.963	0.014
1,8-cineole*	1.03 ± 0.39 a	3.27 ± 0.95 b	0 c	0 c	0 c	205.841	<0.001
(*Z*)-β-ocimene	0.47 ± 0.17 ab	2.37 ± 0.44 a	2.86 ± 1.12 a	2.89 ± 1.25 a	0 b	6.157	0.006
(*E*)-β-ocimene	3.10 ± 0.58 a	15.66 ± 2.97 a	19.77 ± 7.92 a	20.23 ± 8.88 a	0 b	7.883	0.002
γ-terpinene*	3.81 ± 2.08	8.41 ± 0.62	0	0	0	*13.517*	0.009
α-terpineol*	1.17 ± 0.40 a	3.02 ± 0.63 b	0 c	0 c	0 c	319.901	<0.001
silphinene	0 a	0 a	0.27 ± 0.17 a	0.26 ± 0.19 a	3.87 ± 0.45 b	53.674	<0.001
α-copaene*	0 a	0 a	0.94 ± 0.61 b	0.14 ± 0.07 c	0.10 ± 0.02 c	40.954	<0.001
modeph-2-ene	0	0	0.76 ± 0.33	0.88 ± 0.39	9.78 ± 1.10	*15.368*	0.004
α-isocomene*	0 a	0 a	4.84 ± 1.91 b	4.47 ± 1.56 b	34.15 ± 2.94 c	60.626	<0.001
(−)-β-elemene*	0.98 ± 0.40 a	3.15 ± 2.03 a	0 b	0 b	0 b	85.374	<0.001
β-isocomene	0 a	0 a	0.53 ± 0.32 a	0.55 ± 0.27 a	5.86 ± 0.67 b	53.834	<0.001
(−)-(*E*)-β-caryophyllene*	0.72 ± 0.20 a	2.81 ± 0.97 ab	5.49 ± 2.16 bc	1.79 ± 0.53 ac	8.78 ± 0.95 b	10.508	<0.001
β-copaene*	0 a	0 a	0.70 ± 0.31 b	0.10 ± 0.05 c	0.08 ± 0.06 ac	19.326	<0.001
(*E*)-α-bergamotene*	0	0	0	0.23 ± 0.15	0.38 ± 0.02	*12.308*	0.015
(*E*)-β-farnesene*	22.22 ± 7.08 a	245.55 ± 69.35 b	10.63 ± 1.77 a	364.94 ± 72.24 b	209.69 ± 28.48 b	34.133	<0.001
(−)-germacrene D*	5.72 ± 1.31 a	59.68 ± 20.48 bc	126.86 ± 62.58 b	17.56 ± 7.13 ac	0.86 ± 0.13 d	33.412	<0.001
β-selinene*	1.26 ± 0.61 a	6.81 ± 4.15 b	23.98 ± 7.29 c	1.29 ± 0.35 ab	1.60 ± 0.36 ab	19.974	<0.001
bicyclogermacrene	2.25 ± 0.40 ab	22.75 ± 12.82 a	54.74 ± 50.19 a	21.20 ± 18.98 ab	0.22 ± 0.07 b	5.065	0.013
(*E,E*)-α-farnesene*	1.83 ± 0.69 a	5.17 ± 4.16 ab	272.55 ± 83.04 c	9.67 ± 2.72 bd	15.25 ± 3.88 d	29.831	<0.001
germacrene D-4-ol	0 a	0 a	2.51 ± 1.01 b	0.51 ± 0.22 c	0.63 ± 0.14 c	52.965	<0.001
geranyl valerate	0	0	0	0	34.74 ± 4.47	*16.000*	0.003
bisabolol oxide B	78.28 ± 47.30 a	178.26 ± 97.40 a	0 b	0 b	0 b	64.363	<0.001
bisabolone oxide A	5.17 ± 3.22 a	6.73 ± 4.94 a	0 b	0 b	0 b	6.887	0.004
α-bisabolol*	81.48 ± 73.52 a	199.01 ± 171.97 a	0 b	0 b	0 b	17.368	<0.001
bisabolol oxide A	90.34 ± 49.58	320.29 ± 174.17	0	0	0	*14.759*	0.005

Data are given as means ± SE (n = 4). Compounds are listed in order of GC elution time. *Indicates compound identified using authentic standards. All other compounds were tentatively identified based on their mass spectra in comparison to those in the literature and databases as described in the methods. Results of statistical analysis are given as F-values (shown as non-italicized numbers) of a one-way repeated measures ANOVA tests if variances were equal and errors normally distributed, or as χ^2^-values (shown as italicized numbers) if the nonparametric test was used. If the ANOVA revealed significant differences, post-hoc tests were conducted and different letters next to the data indicate significant differences (p < 0.05) among plant organs.

### Isolation of terpene synthase genes from chamomile

**Figure 1:**
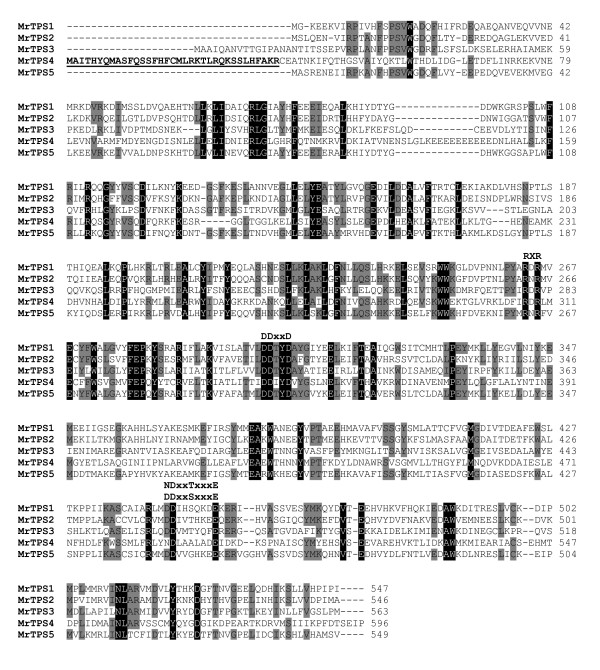
**Alignment of the amino acid sequences of MrTPS1, MrTPS2, MrTPS3, MrTPS4 and MrTPS5.** Amino acids identical in all five proteins are shaded black and amino acids identical in four proteins are shaded gray. The highly conserved metal cofactor binding regions are labeled DDxxD and NDxxTxxxE/DDxxSxxxE, respectively. Amino acids belonging to a predicted signal peptide of MrTPS4 are underlined and shown in bold.

**Figure 2:**
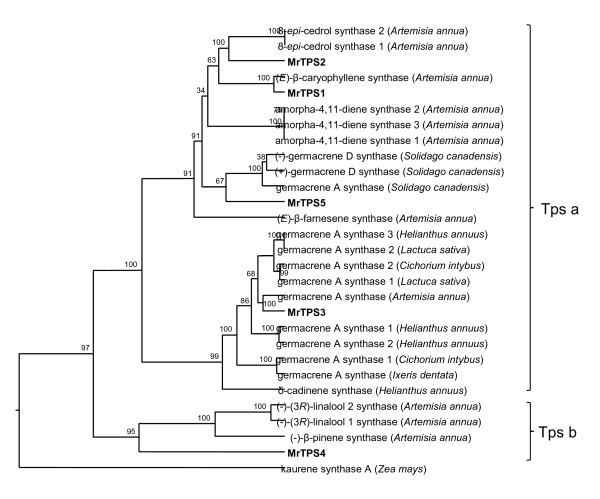
**Phylogenetic tree of terpene synthases from the Asteraceae showing relationships among the isolated chamomile TPS genes.** Clusters corresponding to two TPS subfamilies (TPS a and TPS b) are apparent. As an outgroup, a diterpene synthase from primary metabolism was chosen. New terpene synthases from chamomile are written in bold. The alignment was accomplished with the ClustalX algorithm. Trees were inferred with the neighbor-joining method and n = 1000 replicates for bootstrapping. Accession numbers are provided in Additional file 2: Table S2.

To identify terpene synthase sequences in chamomile we constructed degenerate primers based on known *TPS* genes from other Asteraceae species (Additional file 1: Table S1). Using these primers we could amplify DNA fragments of about 200 bp in length. Sequencing of several of these fragments revealed five different partial *TPS* sequences. The complete open reading frames (ORFs) of these *TPS* genes were obtained by 5′-RACE and 3′RACE. The ORFs with 1638 bp, 1641 bp, 1692 bp, 1791 bp, and 1650 bp were designated as *MrTPS1MrTPS2MrTPS3MrTPS4*, and *MrTPS5*, respectively. The encoded proteins MrTPS1, MrTPS2, MrTPS3, MrTPS4 and MrTPS5 exhibited highly conserved sequence elements of terpene synthases like the DDxxD motif and the NSE/DTE motif, which were implicated in the binding of the substrate diphosphate group (Figure 1). Another conserved sequence found in chamomile terpene synthases was the RxR motif which plays a role in the complexation of the diphosphate group after ionization of the substrate [18]. One of the proteins, MrTPS4, contained an extended N-terminus which was recognized by the program ‘ChloroP’ (http://www.cbs.dtu.dk/services/ChloroP/) as a signal peptide with a length of 35 amino acids (Figure 1). A BLASTP analysis with chamomile TPS in the NCBI protein database (http://www.ncbi.nlm.nih.gov/) revealed that MrTPS1, MrTPS2, MrTPS3, MrTPS4 and MrTPS5 showed highest amino acid sequence similarity to the (*E*)-β-caryophyllene synthase QSH1 (92% similarity) from *Artemisia annua *[19], *epi*-cedrol synthase (63% similarity) from *A. annua *[20], germacrene A synthase CiGASlo (86% similarity) from *Cichorium intybus *[21], (*E*)-β-ocimene synthase (49% similarity) from *Vitis vinifera *[22] and (−)-germacrene D synthase (64% similarity) from *Solidago canadensis *[23], respectively. A dendrogram analysis was conducted to determine the evolutionary relatedness of chamomile terpene synthases to those of other Asteraceae (Figure 2). MrTPS1, MrTPS2, MrTPS3 and MrTPS5 were found to belong to the *TPS a* subfamily which encompasses sesquiterpene synthases from angiosperms, whereas MrTPS4 fell into the *TPS b* subfamily covering angiosperm monoterpene synthases [24]. The Asteraceae *TPS a* sequences formed two distinct clades (Figure 2). MrTPS3 grouped together with different germacrene A synthases and one δ-cadinene synthase in one clade, while MrTPS1, MrTPS2 and MrTPS5 were found in the second clade comprising sesquiterpene synthases with diverse catalytic activities.

### Functional characterization of chamomile terpene synthases

**Figure 3:**
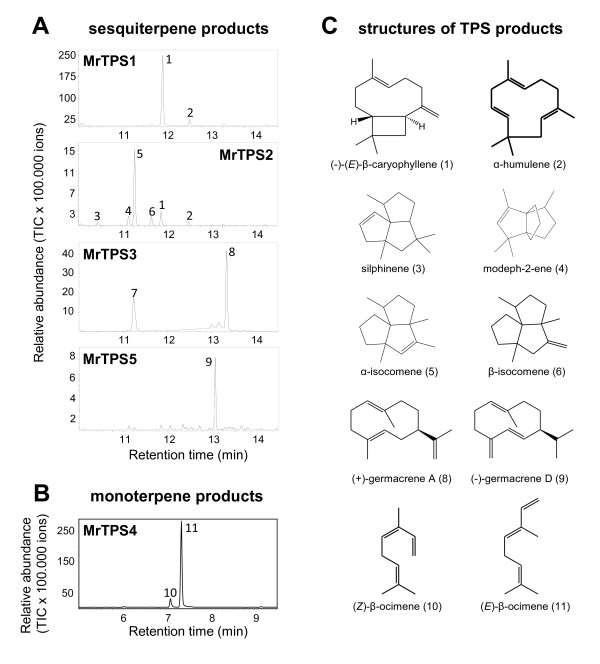
**GC-MS analysis of enzyme products from recombinant MrTPS1, MrTPS2, MrTPS3, MrTPS4, and MrTPS5.** (**A**) Sesquiterpene products from MrTPS1, MrTPS2, MrTPS3 and MrTPS5. (**B**) Monoterpene products from MrTPS4. The enzymes were expressed in *E. coli*, extracted, partially purified, and incubated with the substrates FPP (A) and GPP (B). Products were collected with a solid-phase microextraction (SPME) fiber and analyzed by GC-MS. Analysis of MrTPS3 products was performed using a decreased GC injection temperature (150°C). 1, (*E*)-β-caryophyllene; 2, α-humulene; 3, silphinene; 4, modeph-2-ene; 5, α-isocomene; 6, β-isocomene; 7, β-elemene; 8, germacrene A; 9, germacrene D; 10, (*Z*)-β-ocimene; 11, (*E*)-β-ocimene. Structures of TPS products are presented in (**C**).

For biochemical characterization, the ORFs of chamomile terpene synthases were cloned into the expression vector pASK-IBA7 and the proteins were expressed in *Escherichia coli*. The putative monoterpene synthase MrTPS4 was expressed as a N-terminal truncated protein lacking the predicted signal peptide (Figure 1). Protein extracts from transformed *Escherichia coli* were tested in assays containing the potential substrates geranyl diphosphate (GPP) or farnesyl diphosphate (FPP) in the presence of the cosubstrate Mg^2+^. MrTPS1, MrTPS2, MrTPS3 and MrTPS5 showed highest activity with FPP as substrate and only trace activity with GPP (data not shown) and were therefore characterized as sesquiterpene synthases. MrTPS1 produced (*E*)-β-caryophyllene as the major product and trace amounts of α-humulene (Figure 3A). In contrast, MrTPS2 showed a broader sesquiterpene product spectrum with the angular triquinane, α-isocomene, being the main compound. Additionally, the enzyme produced detectable amounts of three other triquinanes, β-isocomene, silphinene and modeph-2-ene. Like MrTPS1 it also produced (*E*)-β-caryophyllene and α-humulene, but in low amounts (Figure 3A). The major product of MrTPS3 detected under standard GC conditions was β-elemene (data not shown). Since β-elemene can be formed as a thermal rearrangement product from germacrene A in a hot GC injection port [25], MrTPS3 products were also analyzed using a colder GC injector temperature (150°C). Although minor amounts of β-elemene were still present in the GC chromatogram, the major peak could be identified as germacrene A, demonstrating the genuine activity of MrTPS3 (Figure 3A). The enzyme MrTPS5 produced mainly germacrene D and trace amounts of a few unidentified sesquiterpenes. Unlike the other TPS, MrTPS4 only accepted GPP as substrate. It produced acyclic monoterpenes with (*E*)-β-ocimene being the major product and (*Z*)-β-ocimene as a trace compound (Figure 3B). The activity of MrTPS4 confirms the sequence evidence indicating that this enzyme is a monoterpene synthase.

**Figure 4:**
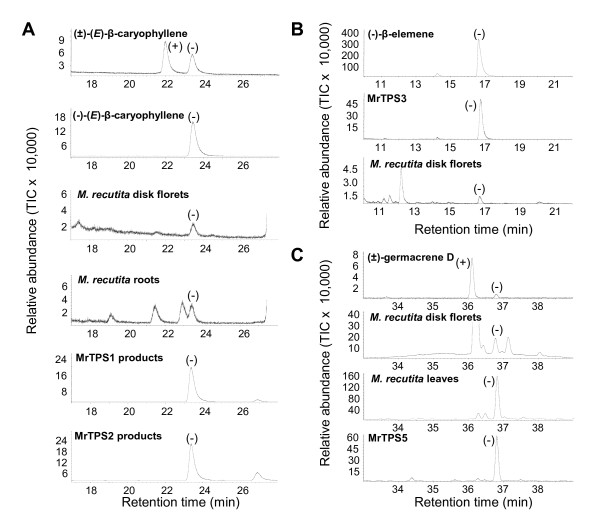
**Chiral analysis of MrTPS enzyme products.** The enzymes MrTPS1/MrTPS2 (**A**), MrTPS3 (**B**) and MrTPS5 (**C**) were expressed in *E. coli*, extracted, partially purified, and incubated with the substrate FPP. Products were analyzed by GC-MS with a chiral column. Retention times and spectra were compared to those of the pure standards (−)-(*E*)-β-caryophyllene, (−)-β-elemene and the standard mixtures (±)-(*E*)-β-caryophyllene and (±)-germacrene D.

A chiral analysis of the enzyme products (*E*)-β-caryophyllene from MrTPS1 and MrTPS2, germacrene A from MrTPS3 and germacrene D from MrTPS5 was performed using chiral GC-MS. Both MrTPS1 and MrTPS2 produced exclusively (−)-(*E*)-β-caryophyllene (Figure 4A). The thermal rearrangement product of germacrene A was identified as (−)-β-elemene (Figure 4B). Since the heat-induced Cope rearrangement of germacrene A to β-elemene retains the stereochemical configuration at C_7 _[25], the enzyme product was determined as (+)-germacrene A. MrTPS5 produced exclusively the (−)-enantiomer of germacrene D. A chiral analysis of (*E*)-β-caryophyllene, β-elemene and germacrene D in the plant extracts revealed that they contained the same enantiomers as produced by the heterologously-expressed enzymes (Figure 4).

### Transcript abundance of *MrTPS* genes in different organs of chamomile

**Figure 5:**
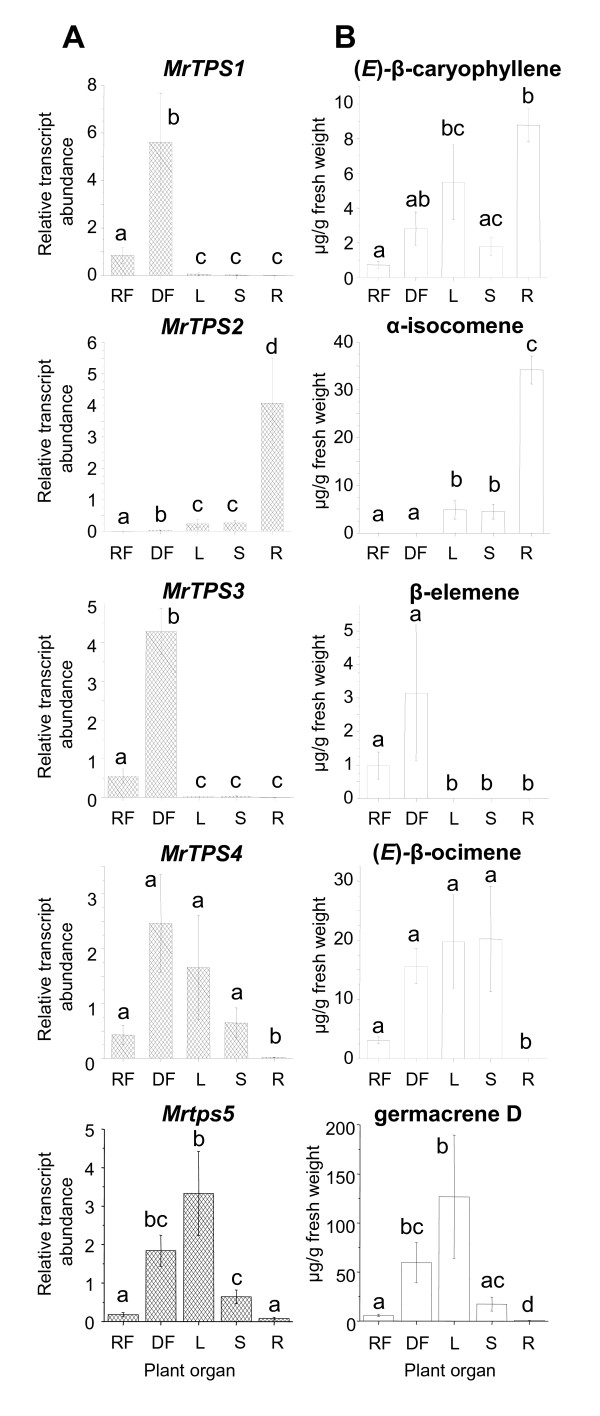
**Transcript accumulation of terpene synthase genes (A) and abundance of single TPS products (B) in different plant organs.** mRNA accumulation of *TPS* genes in different plant organs was measured using qRT-PCR. The relative abundance was determined using a standard curve based method. Terpenes were extracted from plant powder with hexane and analyzed using GC-MS and GC-FID. Bars show means ± SE (n = 4). Different letters indicate significant differences between plant organs. Statistical values for terpene synthase genes: *MrTPS1* – F = 41.916, p < 0.001; *MrTPS2* – F = 49.436, p < 0.001; *MrTPS3* – F = 65.752, p < 0.001; *MrTPS4* – F = 12.037, p < 0.001; *MrTPS5* – F = 30.766, p < 0.001; statistical values for terpenoids see Table 1. RF, ray florets; DF, disk florets; L, leaves; S, stem; R, roots.

To study the *TPS* gene expression in the different plant organs, we measured the accumulation of *TPS* transcripts in the ray florets, disk florets, leaves, stems and roots using qRT-PCR (Figure 5A). *MrTPS1* and *MrTPS3* showed identical expression patterns. The highest transcript accumulation was in the disk florets, a moderate transcript accumulation in ray florets and trace accumulation in the green parts of the plant. Both genes were not expressed in the roots. The monoterpene synthase gene *MrTPS4* and the sesquiterpene synthase gene *MrTPS5* revealed a similar expression pattern with highest transcript accumulation in the green plant parts and the disk florets and only trace transcript accumulation in the roots. In contrast, *MrTPS2* was mainly expressed in roots and only low levels of *MrTPS2* transcripts were also detected in leaves and stems. *MrTPS2* was not expressed in ray florets and showed only trace expression in disk florets.

## Discussion

### Chamomile TPSs contribute to terpene biosynthesis in different organs of the plant

Our analysis confirmed previous studies that showed an organ-specific production of essential oils in chamomile [7]. Such organ-dependent differences in terpene content have also been described for other plant species. For example, the terpene blend of maize leaves is qualitatively and quantitatively different from that of maize roots and maize husks [26]. The composition of such terpene mixtures is often reflected in the summarized product spectra of a few multi-product terpene synthases [13,14,27]. In this study we identified five terpene synthases of chamomile that formed compounds which occur in chamomile oils. The peak expression of *MrTPS3, MrTPS4* and *MrTPS5* in flowers and above-ground tissues corresponded well with the accumulation of their respective enzyme products in these tissues (Figure 5) indicating a direct contribution of MrTPS3, MrTPS4 and MrTPS5 to essential oil biosynthesis in flowers and leaves. The multiproduct enzyme MrTPS2 was mainly expressed in roots and produced some of the sesquiterpenes found in this organ. While *MrTPS1* was only transcribed in flowers, the major enzyme product (*E*)-β-caryophyllene was present in all analyzed plant tissues (Figure 5). Since (*E*)-β-caryophyllene was also formed as a minor product from MrTPS2 it is likely that both MrTPS1 and MrTPS2 are responsible for (*E*)-β-caryophyllene formation in all plant organs. However, the concentration of (*E*)-β-caryophyllene in leaves was higher than expected by the low transcript abundance of *MrTPS1* and *MrTPS2* and could be explained by the presence of another leaf-specific TPS capable of producing this sesquiterpene. Multiple (*E*)-β-caryophyllene synthase genes expressed in different plant organs were also identified in the recently sequenced genome of grapevine [22].

The spatial and temporal production of plant terpenes is often controlled by transcriptional regulation of *TPS* genes. For example, the maize sesquiterpene synthases TPS4 and TPS5 which form the major sesquiterpenes in this plant part are exclusively expressed in the husk covering the maize ears [13]. In Shampoo ginger (*Zingiber zerumbet*), an α-humulene synthase gene was reported to be specifically expressed in the rhizome where it is probably involved in zerumbone biosynthesis [28]. Furthermore, the diurnal emission of the floral monoterpenes myrcene and (*E*)-β-ocimene from snapdragon flowers is controlled by the tissue-specific and rhythmic expression of two monoterpene synthase genes [29]. Posttranscriptional regulation of TPS enzymes [30] as well as light-dependent substrate availability [31] are also discussed as regulatory steps in terpene formation. Our data suggest that the qualitative terpene composition of chamomile essential oils is mainly controlled by the organ-specific expression of *TPS* genes. However, the complete absence of stored monoterpenes in roots could also be explained by the absence of the precursor GPP in that organ. Quantitative differences in terpene content and *TPS* transcript abundance between, for example, ray florets and disk florets could be due to a different density of glandular trichomes, the site of terpene production in these organs.

### MrTPS3 may be the key enzyme in matricine biosynthesis in chamomile flowers

The sesquiterpene lactone matricine is one of the major active compounds of chamomile flowers. It is very unstable and decomposes during steam distillation to chamazulene, a blue compound causing the characteristic color of chamomile oil. Beside the bisabolols and flavonoids, chamazulene is mainly responsible for the antiphlogistic activity of chamomile extracts and oils [1,7].

So far, little is known about the molecular basis of sesquiterpene lactone biosynthesis. The first committed step is the conversion of FPP to the respective sesquiterpene skeletons catalyzed by sesquiterpene synthases. For example, in the Chinese medicinal plant *Artemisia annua*, amorpha-4,11-diene synthase was described as a key enzyme involved in the biosynthesis of artemisinin, an amorphane-type sesquiterpene endoperoxide with antimalarial activity [32-35]. Germacrene A synthases from lettuce (*Lactuca sativa*), chicory (*Cichorium intybus*), *Ixeris dentata* and sunflower (*Helianthus annuus*) were reported to catalyze the first step in guaianolide, eudesmanolide and germacranolide sesquiterpene lactone formation in these plant species [21,36-38].

**Figure 6:**
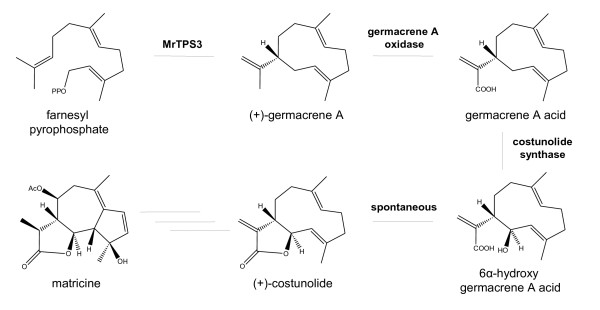
**Proposed biosynthetic pathway for matricine formation in chamomile.** The (+)-germacrene A synthase MrTPS3 is assumed to be the key enzyme in matricine biosynthesis converting the ubiquitous precursor farnesyl pyrophosphate into the basic sesquiterpene skeleton. The reactions forming matricine from (+)-costunolide likely include a cyclization, a hydrogenation of a methyl group, two hydroxylations and a subsequent acetylation. The enzymes catalyzing these reactions are not yet known.

In this study we identified the (+)-germacrene A synthase MrTPS3 from chamomile. The gene was exclusively expressed in disk and ray florets which are known to be the sole accumulation site for the guaianolide sesquiterpene lactone matricine [5,6]. Despite high *MrTPS3* expression levels, only trace amounts of germacrene A were detected in floral tissues as thermally rearranged β-elemene (Table 1, Figure 4). Most likely (+)-germacrene A is rapidly converted into matricine following the proposed reaction path shown in Figure 6. An enzyme that converts germacrene A to germacrene A acid was recently described in lettuce [39]. This protein, a cytochrome P450 of the CYP71 family, catalyzes a regioselective three-step oxidation of (+)-germacrene A at C12. More recently, a second P450 from lettuce, CYP71BL2 was reported to catalyze a 6-α-hydroxylation of germacrene A acid. The resulting 6-α-hydroxy-germacrene A acid spontaneously undergoes a lactonization which yields costunolide, one of the simplest sesquiterpene lactones [40]. The biosynthesis of matricine in chamomile could also follow the route from (+)-germacrene A to costunolide (Figure 6), with further steps including a ring closure forming the guaiane skeleton, two additional hydroxylations, an acetylation and hydrogenation. Although MrTPS3 is likely involved in matricine biosynthesis, we cannot exclude the presence of a further germacrene A synthase which could also contribute to this key reaction. The existence of multiple germacrene A synthases in Asteraceae was recently described for cichory [21] and sunflower [38]. To elucidate the biosynthesis of matricine in more detail, we started to search for other potential germacrene A synthase candidate genes. Furthermore we are planning to identify P450 enzymes in chamomile which might be involved in the formation of costunolide from (+)-germacrene A.

## Conclusions

Terpenes are major components of the essential oils of chamomile and contribute to their pharmaceutical activity. Terpene synthases were identified in this study that are involved in essential oil formation in various plant organs of chamomile. The qualitative terpene composition of the oils seems to be controlled by spatial expression of *TPS* genes. Due to their importance for essential oil production, these genes could be used to generate markers for the breeding of new chamomile cultivars with increased terpene content or with a specific oil composition.

## Methods

### Plant material

Seeds of the Chamomile (*Matricaria recutita*) cultivar ‘Bodegold’ were obtained from Pharmasaat (Artern, Germany). Plants were grown in commercially available potting soil (Tonsubstrat, Klasmann GmbH, Gross-Hesepe, Germany) in a climate-controlled chamber with a 18 h photoperiod, a temperature cycle of 22°C/18°C (day/night) and 65% relative humidity.

For the experiments four plants were used. From each plant disc flowers, ray flowers, leaves, stems and roots were harvested separately. The plant material was immediately ground in liquid nitrogen to a fine powder, which was then used for terpene extractions and molecular work.

### Plant terpene extraction

For terpene extraction, 100 mg of tissue powder was extracted with 400 μl hexane containing 43.2 ng/μl nonyl acetate as an internal standard. The extraction was carried out for 60 minutes at room temperature by vigorous vortexing. The hexane phase was then removed and a 1 μl aliquot was injected into GC-MS for terpene analysis.

### cDNA preparation and RACE library

Total RNA from flowers and roots was isolated using the RNeasy Plant Mini Kit (Qiagen, Hilden, Germany) according to the manufacturer’s instructions. Single-stranded cDNA was synthesized from total RNA using SuperScript™ III Reverse Transcriptase (Invitrogen, Carlsbad, USA) and oligo(dT) primer. A RACE cDNA was constructed from total RNA using the ‘SMARTer™ RACE cDNA Amplification Kit’ according to the manufacturer’s instructions (Clontech, Mountain View, USA).

### Isolation of terpene synthase cDNAs

To isolate fragments of terpene synthase genes, we constructed degenerated oligonucleotides based on conserved sequence elements of other Asteraceae terpene synthases (Additional file 1: Table S1). Using these oligonucleotides, four *TPS* fragments could be amplified from cDNA made from flowers and roots of the chamomile cultivar ‘Bodegold’. The fragments were extended by 5′-RACE and 3′-RACE and the resulting sequences were designated as *MrTPS1*, *MrTPS2*, *MrTPS3*, *MrTPS4*, and *MrTPS5*. The complete open reading frames were amplified from cDNA using the primer pairs TPS1fwd/TPS1rev (*MrTPS1*), TPS2fwd/TPS2rev (*MrTPS2*), TPS3fwd/TPS3rev (*MrTPS3*), TPS4fwd/TPS4rev (*MrTPS4*) and TPS5fwd/rev (*MrTPS5*) (Additional file 1: Table S1). The PCR products were cloned as *Bsa*I fragments into the expression vector pASK-IBA7 (IBA-GmbH, Göttingen, Germany) and several clones were fully sequenced. Sequences were deposited in GenBank (http://www.ncbi.nlm.nih.gov) with the accession numbers JQ255375 (*MrTPS1*), JQ255376 (*MrTPS2*), JQ255377 (*MrTPS3*), JQ255378 (*MrTPS4*), and JQ837261 (*MrTPS5*).

### Sequence analysis

Sequence analysis was performed with the DNASTAR suite of programs (Lasergene, Madison, USA). For dendrogram analysis, the ORFs of terpene synthases were aligned with DNAstar utilizing a clustal W algorithm (matrix: PAM250, gap penalty: 10, gap length: 0.2, delay divergent sequence: 20, DNA transition weight: 0.5) with no additional adjustment. The dendrograms were created using a neighbor-joining algorithm with bootstrap values from 1000 trials.

### Heterologous expression of terpene synthases

For expression, the pASK-IBA7-constructs were introduced into the *E. coli* strain TOP10 (Invitrogen). Liquid cultures of the bacteria harboring the expression constructs were grown at 37°C to an OD_600_ of 0.6. Then, anhydrotetracycline (IBA GmbH) was added to a final concentration of 200 μg/l, and the cultures were incubated for 20 hours at 18°C. The cells were collected by centrifugation and disrupted by a 4 × 30 s treatment with a sonicator (Bandelin UW2070, Berlin, Germany) in chilled extraction buffer (50 mM Tris–HCl, pH 7.5, with 5 mM dithiothreitol and 10% (v/v) glycerol). The cell fragments were removed by centrifugation at 14,000 g and the supernatant was desalted into assay buffer (10 mM Tris–HCl, pH 7.5, 1 mM dithiothreitol, 10% (v/v) glycerol) by passage through a Econopac 10DG column (BioRad, Hercules, USA).

### Assay for terpene synthase activity

To determine the enzymatic activity of the different terpene synthases, enzyme assays containing 40 μl of the bacterial extract and 60 μl assay buffer with 10 μM (*E*,*E*)-FPP or (*E*)-GPP and 10 mM MgCl_2_, in a Teflon-sealed, screw-capped 1 ml GC glass vial were performed. A SPME (solid phase microextraction) fiber consisting of 100 μm polydimethylsiloxane (Supelco, Belafonte, USA) was placed into the headspace of the vial for 0.5 h incubation at 30°C. For analysis of the adsorbed reaction products, the SPME fiber was directly inserted into the injector of the gas chromatograph.

### Gas chromatography – mass spectrometry

Terpene analysis was carried out with a gas chromatograph (Shimadzu model 2010Plus) equipped with a splitless injector (injector temperature, 220°C; injection volume, 1 μl) and coupled to a quadrupole mass selective detector (Shimadzu). H_2_ was used as carrier gas at a flow rate of 1 ml min^-1^. Samples were analysed on a Grace EC™-5 column (30 m × 0.25 mm i.d. × 0.25 μm film thickness, W. R. Grace, Columbia, USA) with a temperature program starting from 50°C for 3 min than increased at a rate of 6°C min^-1^ to 300°C (held for 1 min). For the analysis of MrTPS3 products a colder injector temperature of 150°C was used. The coupled mass spectrometer was operated with a transfer line temperature of 230°C, a source temperature of 230°C, a quadrupole temperature of 150°C, an ionization potential of 70 eV and a scan range of 50–350 amu.

Quantification was performed with the trace of a flame ionization detector (FID) operated at 250°C. Compounds were identified by comparison of retention times and mass spectra to those of authentic reference compounds obtained from Sigma-Aldrich (Steinheim, Germany), Roth (Karlsruhe, Germany), Bedoukian (Danbury, USA) or by reference spectra in the Wiley and National Institute of Standards and Technology libraries and in the literature [41].

Chiral GC-MS analysis was performed on the same instrument using a Rt™-βDEXsm-column (Restek, Bad Homburg, Germany) and a temperature program from 40°C (3 min hold) at 100°C min^-1^ to 100°C (40 min hold). A racemic mixture of (*E*)-β-caryophyllene and a (±)-germacrene D standard prepared from *Solidago canadensis* was kindly provided by Stefan Garms (MPI for Chemical Ecology, Jena, Germany). The (+)-germacrene A synthase CiGASlo from *Cichorium intybus *[21] was used to prepare an authentic (+)-germacrene A standard. *CiGASlo* (Genbank, AF497999) was amplified from cDNA made from total RNA prepared from etiolated chicory heads with the primers GAS1fwd and GAS1rev (Additional file 1: Table S1). The gene was inserted as a *Bsp*MI fragment into the expression vector pASK-IBA7, heterologously expressed and assayed as described above.

### Determination of gene transcript levels

Total RNA was extracted from plant material using the RNeasy kit from Qiagen according to the manufacturer’s specifications. 1,5 μg RNA was DNase treated in a 10 μl reaction using 1 μl DNase from Promega (Madison, USA). 5 μl of DNase treated RNA corresponding to about 0.75 μg of RNA was reverse transcribed in a 20 μl reaction with SuperscriptIII from Invitrogen according to the manufacturer’s specifications using a mix of anchored oligo dT_(12–18)_ and random primers (Invitrogen). To minimize pipetting errors, 5 μl of generated cDNA was used in a 1: 5 dilution as template for qPCR reaction.

Gene-specific primers were designed using Beacon Designer 4 (Premier Biosoft, Palo Alto, USA) with primer length in the range of 19–25 nt, GC content between 40-55% and the amplicon length between 100–300 bp. Primer specificity was confirmed by agarose gel electrophoresis, melting and standard curve analysis and sequence verification of cloned PCR amplicons (see Additional file 1: Table S1 for primer information).

All qPCRs were run as triplicates in 20 μl reactions using the Maxima® SYBR Green QPCR Master Mix from Fermentas (Fermentas GmbH, St. Leon Roth, Germany). Final primer concentration was 0.25 μM. 5 μl of diluted cDNA was used as template. The following PCR protocol was used for all genes: initial incubation at 95°C for 10 min followed by 40 cycles of amplification (95°C for 30 sec, 52-62°C for 30 sec, 72°C for 1 min, plate read), and a melting curve from 52°C to 95°C (0.5°C/5 sec).

Gene expression levels were quantified using the standard curve method. The standard curve was generated using pooled cDNA in equal amounts from all samples. The standard curve was constructed from PCRs using 1 μl, 1/3 μl, 1/9 μl, 1/27 μl in 5 μl as template. All sample expression levels were calculated as multiples of the cDNA pool. *Actin *[42] and *18 S-rRNA *[43] were used as reference genes. Both reference genes showed equal expression levels in the tested plant tissues with ct-value differences of 0.96 (*actin*) and 0.61 (*18 S-rRNA*). The relative expression level in each sample was calculated as the expression level of the respective gene divided by the geometric mean of the expression levels of the two reference genes.

### Statistical analysis

Amounts of terpenoids and relative expressions of *MrTPS* genes are presented as means ± SE. Differences in terpenoid content and relative expression of *MrTPS* genes in different plant organs were analysed by a one-way repeated measures analysis of variance if variances were equal and errors were normally distributed. If these assumptions were not met, data were log-transformed. If the test revealed significant differences between the plant organs (p < 0.05), a post-hoc test (Tukey test) was performed to test for individual differences between the different plant organs. For some terpenoids, variance homogeneity or normality could not be achieved, and so the nonparametric Friedman repeated measures analysis of variance on ranks was conducted. We did not perform post-hoc tests after the nonparametric test since it is nearly impossible to achieve significant differences with a sample size of four. The software package SigmaPlot for Windows version 11.0 (Systat Software Inc. 2008) was used for all analyses.

## Competing interests

The authors declare that they have no competing interests.

## Authors’ contributions

SI and STK carried out the experimental work. GK performed the statistical analysis. JD and JG participated in the design of the study and improved the manuscript. TGK conceived of the study and drafted the manuscript. All authors read and approved the final manuscript.

## Supplementary Material

Additional file 1 **Table S1.** Oligonucleotides used for isolation, cloning and qRT-PCR analysis of MrTPS genes. Click here for file

Additional file 2 **Table S2.** Accession numbers of protein sequences used for dendrogram analysis of Asteraceae TPS. Click here for file
