# Supplementary material for: The organ-specific expression of terpene synthase genes contributes to the terpene hydrocarbon composition of chamomile essential oils
Source: BMC Plant Biol. 2012 Jun 8;12:84. doi: 10.1186/1471-2229-12-84 (PMC3423072; doi:10.1186/1471-2229-12-84)
Supplement: Additional file 1 — Table S1. Oligonucleotides used for isolation, cloning and qRT-PCR analysis of MrTPS genes. [file 1471-2229-12-84-S1.docx]

## Supplemental table 1. Oligonucleotides used for isolation, cloning and qRT-PCR analysis of *MrTPS* genes.

| **name** | **sequence** | **usage** |
| --- | --- | --- |
| Mrdeg-1  Mrdeg-2  Mrdeg-3  TPS1fwd  TPS1rev  TPS2fwd  TPS2rev  TPS3fwd  TPS3rev  TPS4fwd  TPS4rev  TPS5fwd  TPS5rev  GAS1fwd  GAS2rev  TPS1Qfwd  TPS1Qrev  TPS2Qfwd  TPS2Qrev  TPS3Qfwd  TPS3Qrev  TPS4Qfwd  TPS4Qrev  TPS5Qfwd  TPS5Qrev  actQfwd  actQrev  18SQfwd  18SQrev | CCCATBGCVAACTTTYCTCC  AGARATAGADTGGTTGA  CAWAAGCRTCATAHGTGTC  ATGGTACGTCTCAGCGCATGGGGAAAGAAGAGAAAGTGATTC  ATGGTACGTCTCATATCATATTGGTATAGGATGAACGAGCAAA  ATGGTAGGTCTCAGCGCATGTCTTTACAAGAGAATGTTATACG  ATGGTAGGTCTCATATCATGCCATTATAGGATCAACGACAAG  ATGGTACGTCTCAGCGCATGGCAGCCATTCAAGCTAATGTG  ATGGTACGTCTCATATCACATGGGTAGAGAACCCACAAACA  ATGGTACGTCTCAGCGCACCAACAAAATTTTCCAAACCCATG  ATGGTACGTCTCATATCAGGGGATTTCACTAGTATCGAAGG  ATGGTAGGTCTCAGCGCATGGCTTCCAGAGAAAATGAAATTAT  ATGGTAGGTCTCATATCATACACTCATAGCATGAACGAAATGA  ATGGTAACCTGCATTAGCGCATGGCAGCAGTTGAAGCCAACG  ATGGTAACCTGCATTATATCACATGGGTGAAGAACCAACAAACAA  CAGCAGAGGAACATATGGC  CTGGTGGAATACTTTATGGAC  GTGATGCAATTACAGACGAG  GATCACCCGCATGATAACAGG  CACTTCCGCTTATAATGTCATTTC  CTTGGCTTTAGACATCCTTCG  CTTAGACAAAGTGGTTACCG  GCCTCAACATCCTACGATAC  GGATGACACTATGGCAAAGGAG  CGAATCTTCTGACGCTATATCACC  GCTAACAGGGAAAAGATGACTC  ACTGGCATAAAGAGAAAGCACG  ATGA TAACTCGACGGATCGC  CTTGGATGTGGTAGCCGTTT | isolation  isolation  isolation  IBA7  IBA7  IBA7  IBA7  IBA7  IBA7  IBA7  IBA7  IBA7  IBA7  IBA7  IBA7  QPCR  QPCR  QPCR  QPCR  QPCR  QPCR  QPCR  QPCR  QPCR  QPCR  QPCR  QPCR  QPCR  QPCR |

isolation, degenerate primers used for isolation of *TPS* fragments; IBA7, primers used for cloning of the ORF into the vector pASK-IBA7; QPCR, primers used for QRT-PCR analysis.
