# Supplementary material for: The organ-specific expression of terpene synthase genes contributes to the terpene hydrocarbon composition of chamomile essential oils
Source: BMC Plant Biol. 2012 Jun 8;12:84. doi: 10.1186/1471-2229-12-84 (PMC3423072; doi:10.1186/1471-2229-12-84)
Supplement: Additional file 2 — Table S2. Accession numbers of protein sequences used for dendrogram analysis of Asteraceae TPS. [file 1471-2229-12-84-S2.docx]

### Supplemental table 2. Accession numbers of protein sequences used for dendrogram analysis of Asteraceae TPS.

| **name** | **species** | **accession number** |
| --- | --- | --- |
| 8-*epi*-cedrol synthase 1  8-*epi*-cedrol synthase 2  (*E*)-β-caryophyllene synthase  amorpha-4,11-diene synthase 1  amorpha-4,11-diene synthase 2  amorpha-4,11-diene synthase 3  (*E*)-β-farnesene synthase  germacrene A synthase  (-)-β-pinene synthase  (-)-(*3R*)-linalool synthase 1  (-)-(*3R*)-linalool synthase 2  germacrene A synthase 1  germacrene A synthase 2  germacrene A synthase 1  germacrene A synthase 2  germacrene A synthase 3  δ-cadinene synthase  germacrene A synthase  germacrene A synthase 1  germacrene A synthase 2  (+)-germacrene D synthase  (-)-germacrene D synthase  (+)-germacrene A synthase  kaurene synthase A | *Artemisia annua*  *Artemisia annua*  *Artemisia annua*  *Artemisia annua*  *Artemisia annua*  *Artemisia annua*  *Artemisia annua*  *Artemisia annua*  *Artemisia annua*  *Artemisia annua*  *Artemsia annua*  *Cichorium intybus*  *Cichorium intybus*  *Heliantus annuus*  *Heliantus annuus*  *Heliantus annuus*  *Heliantus annuus*  *Ixeris dentata*  *Lactuca sativa*  *Lactuca sativa*  *Solidago canadensis*  *Solidago canadensis*  *Solidago canadensis*  *Zea mays* | AAF80333  CAC08805  AAL79181  CAB94691  AAF61439  AAF98444  AAX39387  ABE03980  AAK58723  AAF13357  AAF13356  AAM21658  AAM21659  ACA14463  ACA33925  ACZ50512  ACA33926  AAL92481  AAM11626  AAM11627  AAR31144  AAR31145  CAC36896  AAA73960 |
